# Hippocampal GluN2A knockdown differentially alters spatial exploration and behavioral performance in male and female rats

**DOI:** 10.3389/fnbeh.2026.1935028

**Published:** 2026-10-06

**Authors:** Maria Florencia Acutain, Fernanda Mariana Silva, Luisina Castellari, María Verónica Baez

**Affiliations:** 1 Laboratorio de Sinapsis y Neurobiología Celular. Instituto de Biología Celular y Neurociencia (IBCN)-CONICET-UBA, Ciudad de Buenos Aires, Argentina; 2 Cátedra de Biología Molecular y Genética. Facultad de Medicina. UBA, Ciudad de Buenos Aires, Argentina

**Keywords:** AAV-shRNA, cognitive flexibility, GluN2A-NMDAR, hippocampal CA1, sex differences, spatial updating

## Abstract

The hippocampus is the brain structure responsible for spatial information processing, map formation, spatially guided behavior, and memory consolidation. Although these processes are often treated as overlapping or even unitary, emerging evidence suggests that they depend on partially dissociable mechanisms. All these processes depend on hippocampal plasticity; more specifically, on NMDA receptor (NMDAR) function and signaling. NMDARs are tetramers containing two obligatory GluN1 subunits and two regulatory subunits whose expression is tightly regulated. Among them, GluN2A, the regulatory subunit mainly expressed in mature structures, is considered a key determinant of synaptic plasticity. Constitutive GluN2A-knockout models have shown impairments in spatial tasks, contextual fear conditioning, and object location memory. However, these models are limited because compensatory mechanisms could be activated during development. To overcome this limitation, we developed an AAV-mediated shRNA knockdown of GluN2A (GluN2A-kd) targeted to the hippocampus in adult Wistar rats, avoiding developmental confounds and enabling the specific assessment of GluN2A function in mature neurons. Our results show that hippocampal GluN2A-kd in adult rats selectively alters spatial exploration and the ability to update spatially guided behavior across tasks, revealing a previously uncharacterized role for GluN2A in the organization of spatial performance rather than in general memory processes. Additionally, we identified a sex-dependent effect of reduced GluN2A expression, suggesting that GluN2A-kd females may exhibit impaired spatial learning or rely on alternative or less efficient spatial strategies to solve the tasks. Together, these findings position GluN2A as a key modulator of the precision and flexibility of spatial behavior required for successful performance in the adult hippocampus.

## Graphical abstract

**Figure:**
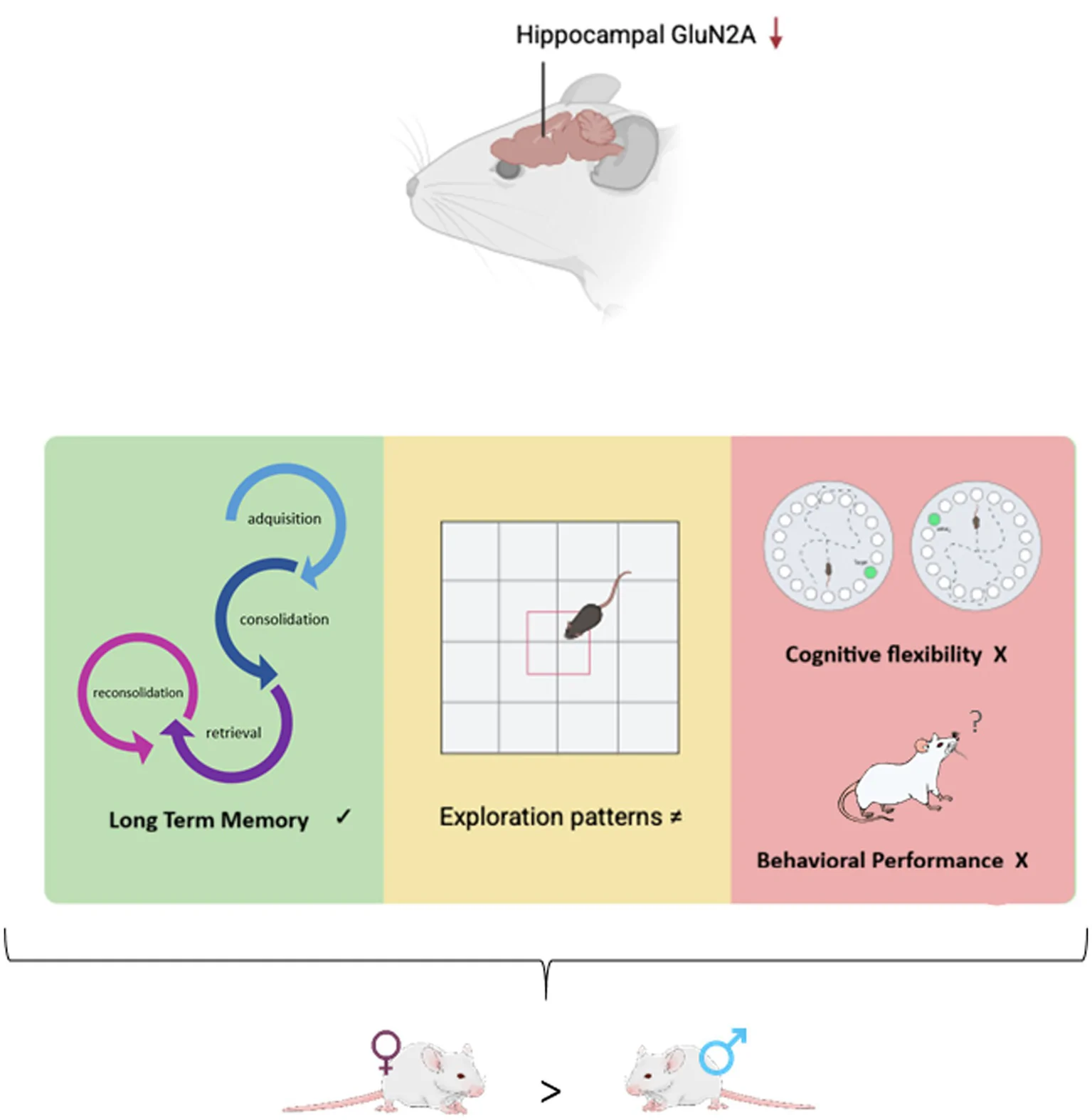
Reduced hippocampal GluN2A expression altered spatial exploration, impaired cognitive flexibility, and reduced behavioral performance, whereas long-term memory remained intact. These effects were more pronounced in females than in males

## 1 Introduction

NMDA receptors (NMDARs) are regulated through complex transcriptional, post-transcriptional, and trafficking mechanisms that determine their abundance and subunit composition across development and brain regions. Among their regulatory subunits, GluN2A and GluN2B (codified by *grin2A* and *grin2B* genes) display a well-defined developmental expression pattern: GluN2B predominates in immature structures, whereas GluN2A becomes enriched after the developmental switch that shapes synaptic maturation in cortical and hippocampal networks (Paoletti et al., 2013; Baez et al., 2018; Ma et al., 2023). This change in subunit receptor composition alters channel kinetics, temporal integration windows, and plasticity thresholds (Paoletti et al., 2013; Sanz-Clemente et al., 2013; Shipton et al., 2014; Ma et al., 2023). For this reason, GluN2A is considered a key determinant of synaptic plasticity in mature circuits.

*Grin2A* knockout (KO) mice did not show developmental delay nor developmental deficits (Bannerman et al., 2008; Gray et al., 2011; Bertocchi, 2021; Hosseini et al., 2025). However, decreased GluN2A expression was associated with different cognitive impairments (Amador et al., 2020; Franchini et al., 2020; Acutain et al., 2021; Du et al., 2021; Hosseini et al., 2025), and more recently, reduced GluN2A expression was related to schizophrenia (SCZ) related symptoms (Alsabban et al., 2020; Farsi et al., 2023; Herzog et al., 2023; Hosseini et al., 2025). Several authors have reported that reduced GluN2A expression impairs the contextual component of the fear-conditioning paradigm (Kiyama et al., 1998; Ng et al., 2009; Franchini et al., 2019; Acutain et al., 2021; Gao et al., 2022) or the acquisition of a new location of a familiar object (Ng et al., 2009; Franchini et al., 2019). Accordingly, indirect reduction of GluN2A expression in Neto1 KO mice led to deficits in cognitive flexibility, although acquisition of the Morris water maze task (MWM) remained intact (Ng et al., 2009). Also, Alsabban and collaborators indicated that indirect GluN2A reduction due to kif3b^+/−^ or kif3b-KO induced a deficit in social interaction and a poor spatial memory (Alsabban et al., 2020). In parallel, silencing GluN2A in astrocytes of Wistar rats was associated with increased latencies to locate the escape hole in the Barnes maze (BM) (Du et al., 2021). More recently, Hosseini et al. reported that grin2A^+/−^ mice showed impaired working memory performance in the radial arm maze, while reference memory errors were comparable to controls (Hosseini et al., 2025).

These studies of GluN2A function rely on constitutive or global loss-of-function models (Kiyama et al., 1998; Bannerman et al., 2008; Ng et al., 2009; Franchini et al., 2019; Alsabban et al., 2020; Du et al., 2021; Gao et al., 2022). Because GluN2A silencing occurs since early development, it is difficult to determine whether the observed deficits arise from developmental alterations or from the role of GluN2A in fully mature structures where this subunit is relevant.

A fundamental gap in the field concerns how reducing GluN2A expression in an already mature hippocampal circuit influences spatial information processing, map formation, spatially guided behaviors and memory consolidation. This distinction is essential because spatial memory is often treated as a unitary construct, even though growing evidence indicates that the formation of a spatial map and the behavioral use of learned information may rely on partially dissociable mechanisms within the hippocampus (Morris et al., 1982; Mumby et al., 2002; Place et al., 2012; Redondo et al., 2014; Maier et al., 2024). To address this hypothesis, we used an adeno-associated vector (AAV)-mediated shRNA knockdown of GluN2A (GluN2A-kd) targeted to hippocampus after the GluN2B/GluN2A switch (Acutain et al., 2021), thereby avoiding developmental confounding and enabling the specific assessment of GluN2A function in mature circuits. This approach allowed us to evaluate how reduced GluN2A expression affects spatial exploration and updating spatial behavior when contextual information changes. Our results show that hippocampal GluN2A-kd in a mature circuit selectively altered spatial exploration and behavioral flexibility across tasks, revealing a previously uncharacterized role for GluN2A in the accuracy of spatially guided behavioral performance. Additionally, we identify a sex-dependent effect, with females showing more pronounced behavioral alterations under reduced GluN2A expression. Although one possible explanation is that females may rely on distinct or less efficient spatial strategies, impaired task learning cannot be ruled out with the present design.

## 2 Materials and methods

### 2.1 Animals

Male (n = 20, Weight = 290–320 g) and female (*n* = 20, Weight = 200–250 g) Wistar rats from the IBCN Animal Facility were used. The rats were maintained under controlled environmental conditions (22 ± 2 °C; 65% humidity) with a 12:12 h light/dark cycle. Animals were housed in Plexiglas cages (four per cage) with food and water provided *ad libitum*.

All experimental procedures were conducted in accordance with the U. S. National Institutes of Health Guide for the Care and Use of Laboratory Animals and approved by the Animal Care and Use Committee of the University of Buenos Aires (CICUAL, Res. Number RESCD-2023-3685-E-UBA-DCT#FMED). All efforts were made to reduce the number of animals used and to minimize suffering. Methods and results are reported according to the revised ARRIVE guidelines (Percie du Sert et al., 2020).

### 2.2 Surgery and viral vector administration

The surgical procedure was performed as previously described (Acutain et al., 2021). Rats were anesthetized by intraperitoneal injection of ketamine (70 mg/kg) and xylazine (10 mg/kg). Adequate anesthesia was confirmed by loss of pedal withdrawal and corneal reflexes prior to the procedure. Animals were monitored throughout to ensure maintenance of surgical anesthesia. All procedures complied with ARRIVE 2.0 guidelines (Percie du Sert et al., 2020) and institutional animal-care regulations (CICUAL, Res. Number RESCD-2023-3685-E-UBA-DCT#FMED). Adeno-Associated viral vectors: AAV-shGluN2A or AAV-shsc (MOI: 2 × 10^4^; 2 μL per side) were bilaterally injected into the CA1 region of the dorsal hippocampus (AP − 4.0 mm, ML ± 3.0 mm, DV − 1.8 mm; from Bregma); (Paxinos and Watson, 2007) (Acutain et al., 2021). Immediately after surgery, rats received meloxicam (1 mg/kg once every 24 h for 72 h) for analgesia. After surgery, rats were returned to their home cages with food and water *ad libitum* animals were monitored constantly during the first 4–6 h and then at least twice daily for 72 h. Monitoring included assessment of general activity (locomotion, exploration, social interaction), grooming, posture, and responsiveness to handling. The surgical site was inspected for signs of inflammation, discharge, bleeding, or infection. Body condition, hydration status, and food and water intake were also checked. Any animal showing signs of pain or distress beyond expected postoperative discomfort was evaluated by animal facility staff and treated accordingly. No signs of suffering were observed in these analyses or during behavioral testing. Two GluN2A-kd rats were excluded from all analyses because their GluN2A expression levels were comparable to those of control animals. Additionally, one female and one male SC rat were excluded from specific behavioral analyses due to experimental conditions detailed in the Supplementary Table S1.

### 2.3 Behavioral assays

14 days post-injection (DPI), animals were injected with the AAV-shSc (controls, Sc; n = 10/sex) or AAV-shGluN2A (GluN2A-kd, 2A; n = 10/sex). The number of rats per group was chosen to balance ethical and practical considerations with the need to detect biologically relevant effects and was in line with previous studies in the field (Baez et al., 2013; Cercato et al., 2017; Acutain et al., 2021). Animals were assigned to groups using block randomization by surgery day (7–10 animals per block). Within each block, animals were allocated to the control or GluN2A-kd group using a predetermined alternating sequence as they entered surgery. This procedure produced balanced group sizes and mixed group composition within cages (typically 2:2, occasionally 3:1). Each block began behavioral testing on a different start day.

Rats were subjected to the following battery of behavioral tasks: new object location (NOL), adapted three-chamber social test (A3CST), two-trial social memory test (2TSMT), direct Barnes maze (DBM) and reverse Barnes maze (RBM) (Figure 1). These assays were conducted daily starting 1 h after lights on and continuing for approximately 5 h. All the behavioral tasks were recorded and analyzed offline by researchers blinded to the treatment. One day after finalizing behavioral assays, rats were euthanized by gradual-fill CO₂ inhalation followed by decapitation, in accordance with the AVMA Guidelines for the Euthanasia of Animals (Leary, 2020) and the CA1 region of both hippocampi from each rat were microdissected to analyze *grin2A* levels.

**Figure 1:**
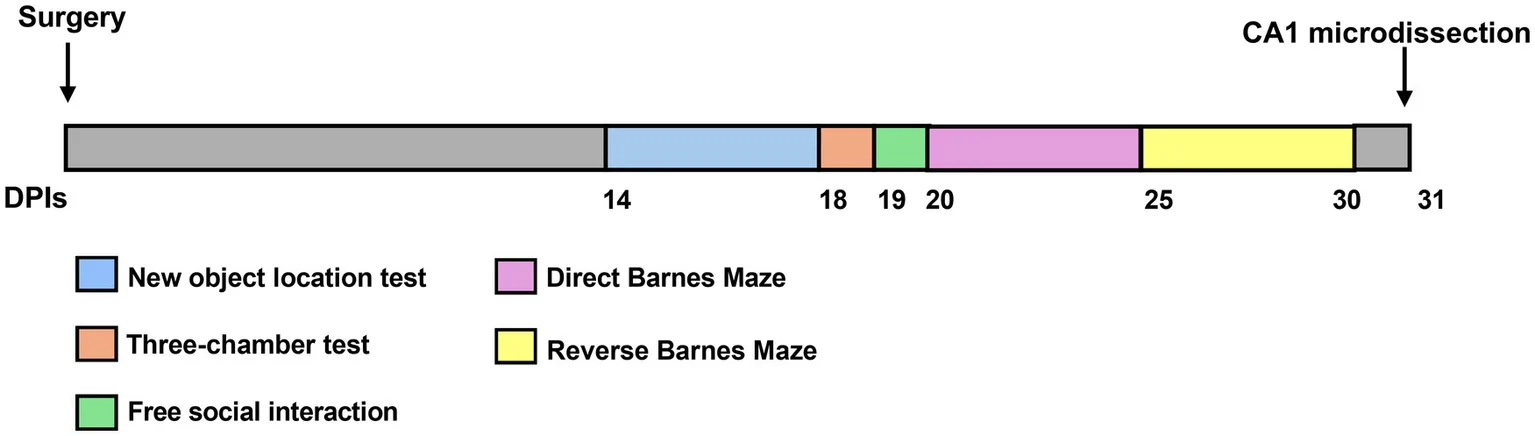
Experimental design. Male and female rats were injected bilaterally with AAV-shSc (Sc, *n* = 10/sex) or AAV-shGluN2A (2A, *n* = 10/sex) in the hippocampal CA1 region. After 14 DPI, the four groups were subjected to the following tasks: New object location (NOL), adapted three-chamber social test (A3CST), two-trial social memory test (2TSMT), direct Barnes maze (DBM), and reverse Barnes maze (RBM). One day after the end of the behavioral battery (31 DPIs) animals were euthanized, and the hippocampi collected to perform mRNA extraction.

### 2.4 Object location task (NOL)

As a first task, the four groups of animals (controls and GluN2A-kd; females and males) were evaluated in a NOL task composed of four sessions. During the first two sessions, control and GluN2A-kd rats were exposed to an open field (OF; 80 cm × 80 cm x 80 cm) for 10 min per day, over two consecutive days, to habituate them to the arena (Figure 2A). On the third day, two identical objects were placed in the OF at equidistant positions (training session), and rats were allowed to explore the arena and the objects for 5 min. On the following day, one of the objects was displaced to a new location (test session), and animals were, again, allowed to explore the objects and the arena for 5 min. After each session, rats were returned to their home cages. All sessions were recorded, and the time spent exploring each object was quantified. A discrimination index (DI) was calculated using the following formula:


$$\mathrm{DI}=\frac{\mathrm{Tim}e_{\text{Novel position}}-\mathrm{Tim}e_{\text{Familiar position}}\;}{\mathrm{Tim}e_{\text{Novel position}}+\mathrm{Tim}e_{\text{Familiar position}.}\;}$$

**Figure 2:**
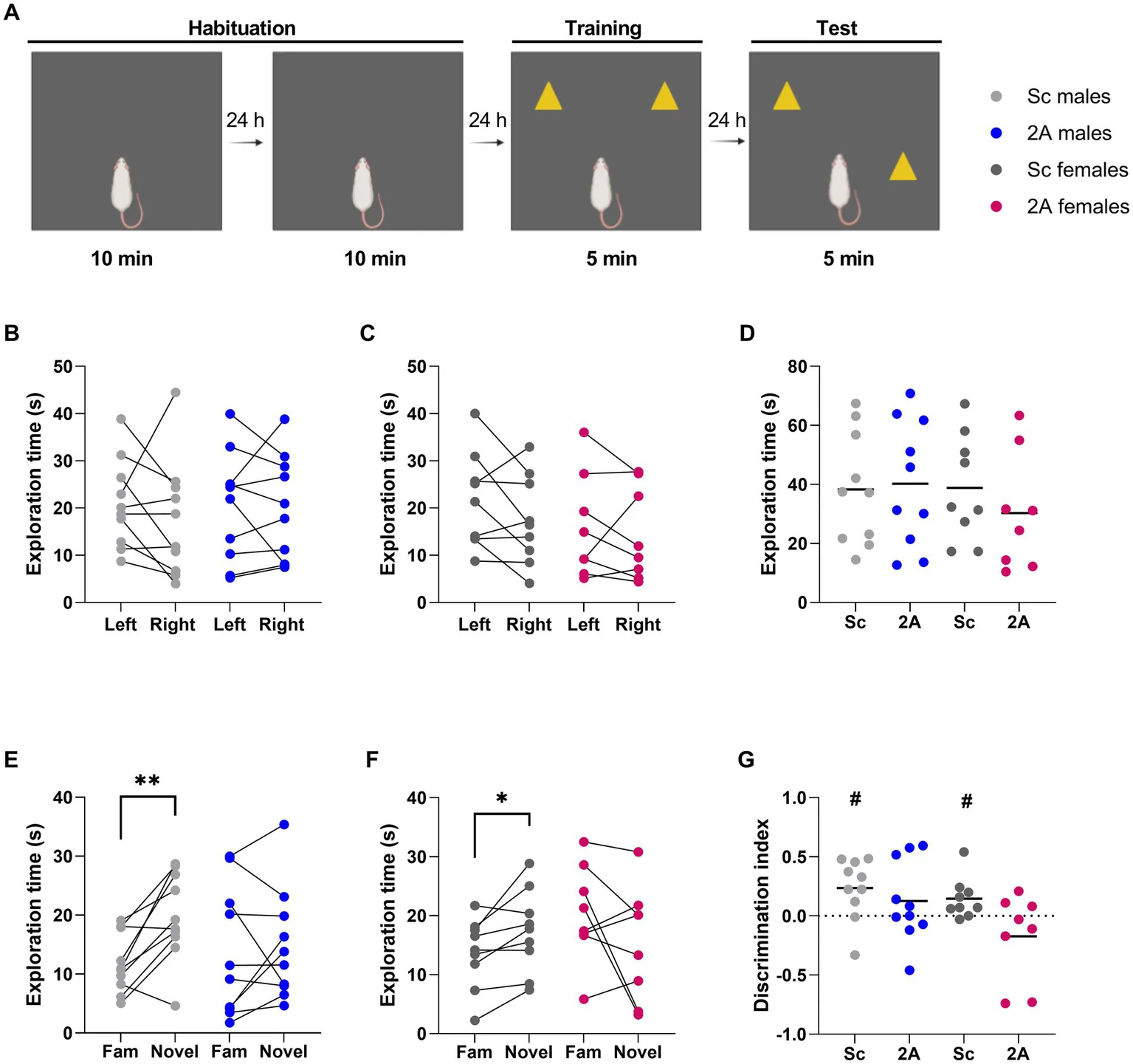
GluN2A-kd rats showed a deficit in NOL task. **(A)** Schematic diagram of the NOL paradigm. **(B,C)** Time spent exploring right and left objects during training for control (Sc) and GluN2A-kd (2A) rats. **(B)** Males (light gray and blue dots respectively). **(C)** Females (dark gray and pink dots). None of the four groups showed significant differences between right and left objects. Sc-males *p* = 0.3414, 2A-males *p* = 0.8262; Sc-females *p* = 0.1609, 2A-females *p* = 0.5823; paired *t* test. **(D)** Total exploration time during the training session. Non-significant differences were observed between groups. Two-way ANOVA; factors: Group and Sex; Group: % of variance: 0.7471, *p* = 0.6159; Sex: % of variance: 1.547, *p* = 0.4713; Interaction: % of variance 1.892, *p* = 0.4261. **(E,F)** Time exploring each object in the familiar (Fam) or novel (Novel) position during the test session. Only Sc-males **(E)** and Sc-females **(F)** explored the novel position significantly longer than the familiar one Sc-males ** *p* = 0.0037; 2A-males *p* = 0.6495; Sc-females * *p* = 0.0239, 2A-females *p* = 0.1727; paired *t* test. **(G)** Sc-male and Sc-female rats showed DI values significantly different from zero whereas GluN2A-kd rats did not. Sc-males # *p* = 0.0176, Sc-females # *p* = 0.0353, 2A-males *p* = 0.2773, 2A-females *p* = 0.2263; one sample *t* test. There are significant differences between Sc- and 2A-rats (two-way ANOVA; factors: Sex and vector; sex: % of variance: 9.461, *p* = 0.0536; vector: % of variance: 11.57, * *p* = 0.0339; Interaction: % of variance: 2.736, *p* = 0.2897). Sc-males, *n* = 10; 2A-males, *n* = 10; Sc-females, *n* = 9; 2A-females, *n* = 8.

### 2.5 Adapted three-chamber social test (A3CST)

The three-chamber social test was adapted from previously described protocol (Yang et al., 2011) to encourage exploration of the cages rather than the open space. The three-chamber apparatus consisted of an arena (50 × 50 × 39 cm) containing two cages (30 cm × 20 cm × 15 cm) (Figure 3A). The test consisted of three 5-min sessions separated by 5-min intersession intervals. Animals were isolated for 30 min prior to the task in order to promote social exploration and were returned to their individual cages during each intersession period. In the first session, the experimental animal (Re) was habituated in the arena and allowed to freely explore both the space and the empty cages. In the second session, a novel, younger, same-sex rat (R1) was placed in one of the cages, while an object was placed in the other, to assess social interest. In the third session, the object was replaced with a second novel, younger, same-sex rat (R2), allowing comparison between the familiar animal (R1) and the novel one (R2) to evaluate social novelty. The time that Re spent exploring the cages, the object, R1, and R2 was recorded, and the DIs were calculated using the following formulas:


$$DI_{R1-O}=\frac{\mathrm{Tim}e_{R1}-\mathrm{Tim}e_{O}}{\mathrm{Tim}e_{R1}+\mathrm{Tim}e_{O}}$$

$$DI_{R2-R1}=\frac{\mathrm{Tim}e_{R2}-\mathrm{Tim}e_{R1}}{\mathrm{Tim}e_{R2}+\mathrm{Tim}e_{R1}}$$

**Figure 3:**
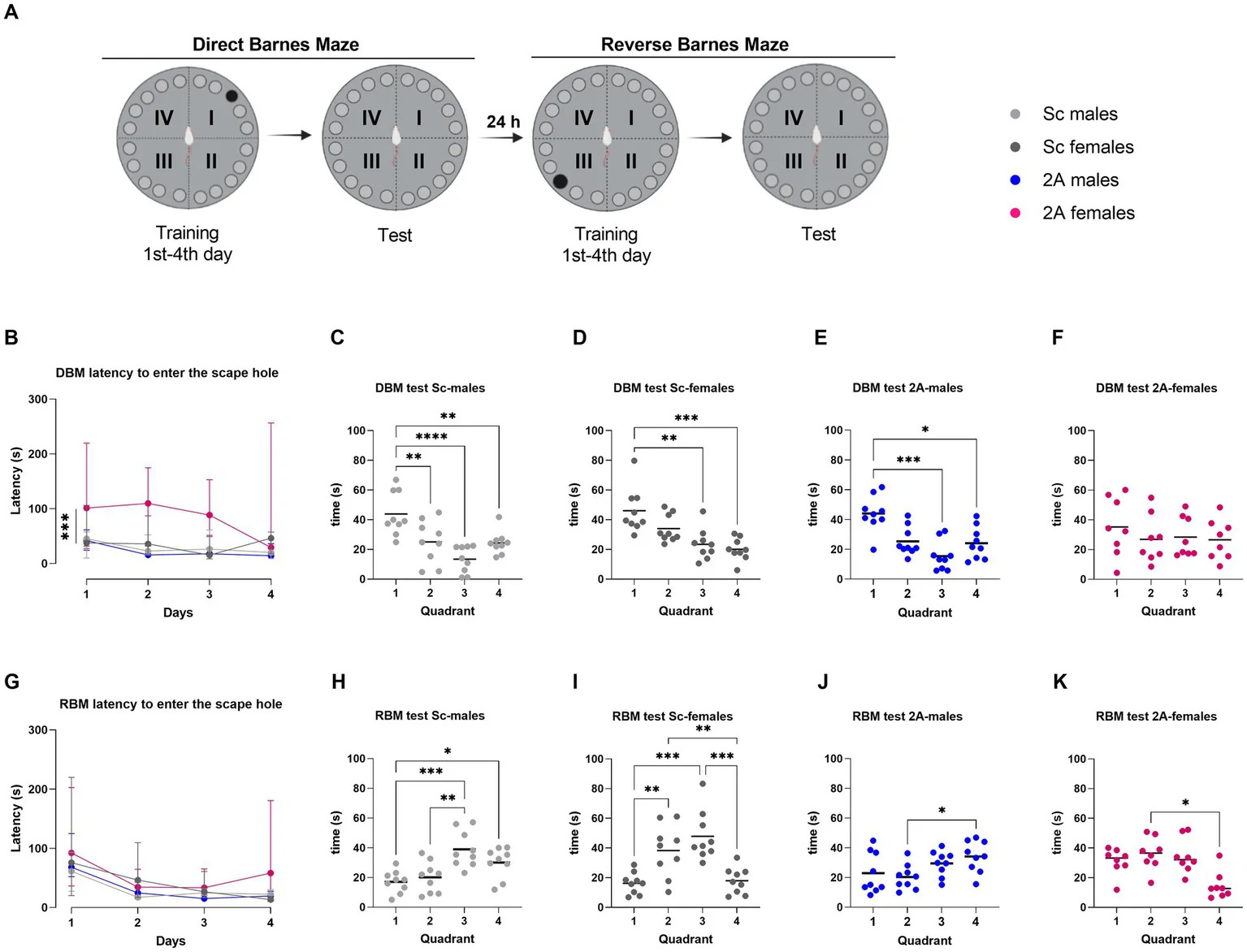
GluN2A-kd rats learn a multisession task but fail to show cognitive flexibility. **(A)** Schematic diagram of the Barnes maze task. **(B)** Latency to escape from the platform during DBM 4 days training. Sc-males (light gray line), Sc-females (dark gray line), and 2A-males (blue line) showed a decrease in their escape latency throughout the fur training days. In contrast, 2A female rats (pink line) did not. (*** *p* = 0.0004, two-way ANOVA; * *p* = 0.0168 2A- vs. Sc-females, *** *p* = 0.0004 2A-females vs. Sc-males, *** *p* = 0.0001 2A-males vs. 2A-females; Tukey’s multiple comparison test) **(C–F)**. Time exploring each quadrant in the DBM test session. **(C)** Sc-males, light gray dots. **(D)** Sc-females, dark gray dots. **(E)** 2A-males, blue dots. **(F)** 2A-females, pink dots. Sc-rats as well as male 2A-rats explore significantly Q1 longer than the opposite one (Q3). However, 2A female rats explored similar amount of time all quadrants. Sc-males **** *p* < 0.0001, 2A-males **** *p* < 0.0001; Ordinary one-way ANOVA; Sc-females ** *p* = 0.0031, 2A-females *p* = 0.6429, Kruskal-Wallis test; Sc-males Q1 vs. Q3 **** *p* < 0.0001, Q1 vs. Q2 ** *p* = 0.0079, Q1 vs. Q4 ** *p* = 0.0072; 2A-males Q1 vs. Q3 **** *p* < 0.0001, Q1 vs. Q2 ** *p* = 0.0031, Q1 vs. Q4 ** *p* = 0.0020; Holm-Sidak’s multiple comparisons test; Sc-females Q1 vs. Q3 * *p* = 0.0219, Q1 vs. Q4 ** *p* = 0.0069; Dunn’s multiple comparisons test. **(G)** Latency to escape from the platform during RBM training. The four groups exhibited short and similar latencies (*p* = 0.1899, two-way ANOVA) **(H–K)** Time exploring quadrants during the RBM test session. **(H)** Sc-males. **(I)** Sc-females. **(J)** 2A-males. **(K)** 2A-females. Only Sc-rats exhibited cognitive flexibility and spent more time exploring the target quadrant (Q3). (Sc-males *** *p* = 0.0002; Sc-females **** *p* < 0.0001, ordinary one-way ANOVA; Sc-males Q3 vs. Q1 *** *p* = 0.0004, Q3 vs. Q2, ** *p* = 0.0021, Q1 vs. Q4 * *p* = 0.0435; Sc-females Q3 vs. Q1 *** *p* = 0.0003, Q1 vs. Q2 ** *p* = 0.0096, Q2 vs. Q4 ** *p* = 0.0096, Q3 vs. Q4: *** *p* = 0.0003; Holm-Sidak’s multiple comparisons. 2A-male * *p* = 0.0347, Ordinary one-way ANOVA; Q3 vs. Q1 *p* = 0.4651; Q2 vs. Q4 * *p* = 0.0499; Holm-Sidak’s multiple comparisons test. 2A-female ** *p* = 0.0093, Kruskal-Wallis test; Q3 vs. Q1: *p* > 0.9999; Q2 vs. Q4 * *p* = 0.0109; Dunn’s multiple comparisons test). Sc-males, *n* = 9; 2A-males, *n* = 9; Sc-females, *n* = 9; 2A-females, *n* = 8.

### 2.6 Two trial social memory test (2TSMT)

To assess social interaction and social novelty in freely moving rats, we used a protocol adapted from a previous work (Leser and Wagner, 2015). In the first session (Figure 4A), the experimental rat (Re) was habituated to an arena (80 × 80 × 80 cm) for 2 min. Immediately afterward, a younger, unfamiliar, same-sex rat (R1) was introduced into the arena, and both animals were allowed to freely interact for 5 min. Both Re and R1 were then returned to their individual cages for 40 min, and the arena was cleaned. In the second session, the Re and the R1 (familiar rat) were reintroduced into the arena together with a new, younger, same-sex rat (R2, novel rat) matched to the familiar one. The three animals were allowed to freely interact for 5 min, after which they were returned to their home cages. The DI for this phase was calculated as the DI_R2-R1_ in the A3CST as follows:


$$DI_{R2-R1}=\frac{\mathrm{Tim}e_{R2}-\mathrm{Tim}e_{R1}}{\mathrm{Tim}e_{R2}+\mathrm{Tim}e_{R1}}$$

**Figure 4:**
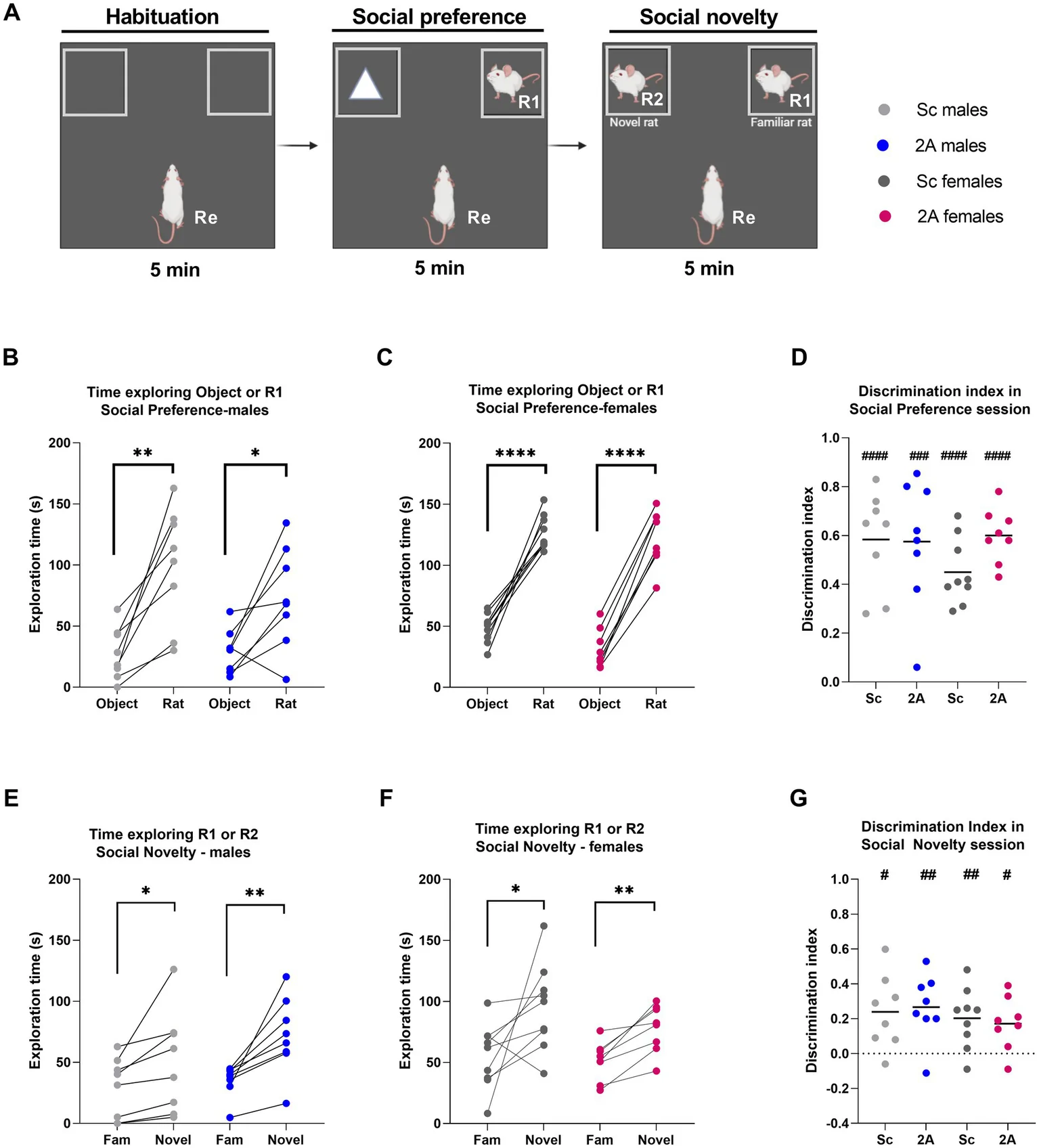
GluN2A-kd rats exhibit conserved social interaction and recognition. **(A)** Experimental scheme of the A3CST. **(B,C)** Time exploring the object or the rat during social preference session. **(B)** Sc- and 2A-males (light gray and blue dots respectively). **(C)** Sc- and 2A-females (dark gray and pink dots, respectively). The four groups explored the R1 significantly more than the object (Sc-males ** *p* = 0.0021, 2A-males * *p* = 0.0194, Sc-females **** *p* < 0.0001, 2A-females **** *p* < 0.0001, Paired t test). **(D)** DI values for the social preference session. All DI values were significantly different from zero (Sc-males #### *p* < 0.0001, 2A-males ### *p* = 0.0004, Sc-females #### *p* < 0.0001, 2A-females #### *p* < 0.0001, One sample t test). (Sc vs. 2A *p* = 0.9164, females vs. males *p* = 0.7717, Two-way ANOVA). **(E,F)** Time exploring the novel (R2) or the familiar rat (Fam, R1) during social recognition session. The four groups: **(E)** Sc- and 2A-males and **(F)** Sc- and 2A-female rats explore the novel rat (R2) longer than the familiar one (R1) (Sc-males * *p* = 0.0395, 2A-males ** *p* = 0.0046, Sc-females * *p* = 0.0483, 2A-females ** *p* = 0.0011; Paired t test). **(G)** DI for the social preference session. DI values were significantly different from zero for the four groups (Sc-males # *p* = 0.0152, 2A-males ## *p* = 0.0055, Sc-females ## *p* = 0.0076, 2A-females # *p* = 0.0153, One sample t test). (Sc vs. 2A *p* = 0.3090, females vs. males *p* = 0.9787, Two-way ANOVA). Sc-males, *n* = 8; 2A-males, *n* = 8; Sc-females, *n* = 9; 2A-females, *n* = 8.

Before testing, each Re was individually housed for 30 min to promote social exploration.

### 2.7 Barnes maze (BM) task

The BM used for this work consists of a black octagonal platform 120 cm diameter, elevated 100 cm above the ground. The platform contained 20 evenly spaced holes, with a removable escape box positioned beneath the designated target hole. A second removable box (start box) was used to place each rat in the center of the maze at the beginning of each trial. The maze was positioned at the center of the experimental room and illuminated to provide a mildly aversive context that encouraged rats to locate the escape box. A video camera mounted above the apparatus recorded each daily session. Videos were analyzed using Tox-Trac software (version 1.1, 2025; RRID: SCR_021502) (Rodriguez et al., 2018) to quantify exploration patterns. The latency to escape from the platform was monitored and scored for each trial. During test sessions analysis, the maze was divided into four quadrants, with the division arranged so that the target hole was centered within Quadrant 1 (Q1). The time spent in each Q was quantified. Learning was considered successful when the rat spent more time in the target quadrant than in the opposite quadrant.

On the first day, prior to training, animals were habituated in the escape box for 30 s. For each training session, rats were placed in the center of the maze inside the start box for 10 s. Then, each animal was allowed to explore the maze until they entered the escape box. If a rat failed to locate the escape hole within 3 min, it was gently guided to the target. After entering the escape box, rats remained inside for 30 s before returning to their home-cage. All animals underwent one training session per day for four consecutive days. On the fifth day, spatial long-term memory was assessed in a test session in which the escape hole was removed. Each rat was placed in the center of the maze and allowed to explore it for 2 min, after which it was returned to its home cage.

The day following direct BM (DBM), we started to train the rats in the reversal phase of the BM (RBM). This phase was performed using the same protocol as the DBM changing the escape hole 180° from its original position.

To avoid potential spatial bias and ensure balanced goal locations across subjects, the escape hole was placed in different positions. After each session, all maze surfaces were cleaned with 70% ethanol and allowed to dry.

### 2.8 Quantitative real-time PCR (RT-qPCR)

Total RNA from microdissected CA1 hippocampal region of each rat was extracted in ARNPrepZOL reagent (Inbio Highway, Argentina) following manufacturer’s instructions. The isolated RNA of each sample was dissolved in RNase-free water, and RNA concentration was determined spectrophotometrically using NanoDrop Lite Plus instrument (Thermo Scientific, USA). Complementary DNA (cDNA) synthesis was performed according to the manufacturer’s instructions using 1 μg of total RNA as template, 200 pmol random hexamer primers (Genbiotech, United States), 100 nmol dNTP mix (Cat. No. PD2001, Genbiotech, United States), 20 U RNAsin (Cat. No. PE3011, Genbiotech, United States), and 200 U MMLV reverse transcriptase (Cat. No. M1303, Sigma-Aldrich, United States). The resulting cDNA samples were stored at −20 °C until analysis.

Quantitative real-time PCR amplification was performed on AriaMx Real-Time PCR System (Agilent Technologies, United States). The reaction was carried out as previously described (Acutain et al., 2021), using SYBR® Select Master Mix (Cat. No. 3353805, Applied Biosystems, Life Technologies, United States) and 10 pmol of each reported primer for *grin2a* (NM_012573.3 — Fw: CCCAGGCTTGTGGTGATCGT; Rv: CGAAGGGGGCTTCCTCCAAG) or *cypA* (NM_017101.1 — Fw: TATCTGCACTGCCAAGACTGAGTG; Rv: CTTCTTGCTGGTCTTGCCATTCC) detection (Acutain et al., 2021). GluN2A mRNA expression was calculated by relative standard curve approach by using Ct values and amplification efficiencies obtained with AriaMx software (Version 2.0, RRID: SCR_019469, Agilent Technologies). Negative controls were included in every assay, and amplification specificity was confirmed through melting-curve analysis.

### 2.9 Statistical analysis

For each variable, normality was assessed using the Kolmogorov–Smirnov test, followed by an F test to compare variances and assess homoscedasticity. When variables passed both tests, Student’s t test was applied to compare two groups. For more than two groups, one-way ANOVA followed by Tukey’s post hoc test was applied. For comparisons involving vector and sex, two-way ANOVA followed by Tukey’s post hoc test was used.

In the case of discrete and non-Gaussian variables, the Mann-Whitney or Kruskal-Wallis, followed by Dunn’s multiple comparisons test, were applied for two or more variables, respectively. In some cases, when variables were compared with a constant (= 1), a one-sample t test was applied. In all cases, individual values corresponding to each rat were plotted. Significance levels are shown in the text and the corresponding figure legend. Statistical parameters are reported in the Supplemental Table. All analyses were performed using GraphPad Prism version 9.2 (RRID:SCR_002798).

For correlation analysis, simple regression analysis was applied by using R-Studio (RRID: SCR_000432), indicating the R^2^ and *p* values.

Exploration patterns were analyzed by Markov series with R-Studio. For global differences between DBM vs. RBM, Females vs. males and sc vs. 2a, Fisher’s exact test was applied. For differences between transitions in sc vs. 2a, only transitions with valid contingency tables were tested by Wilcoxon Test. Transitions that did not meet these criteria were excluded from statistical comparison. Heatmaps were generated to visualize transition probabilities and group differences.

## 3 Results

### 3.1 GluN2A-kd rats show an impairment in one-session training task

Given that GluN2A knockdown was specifically induced in the hippocampus, and previous findings have demonstrated that GluN2A-kd rats exhibit impairments in the contextual component of fear conditioning tasks (Acutain et al., 2021), we initiated our behavioral characterization by performing a NOL paradigm test (Figure 1, Figure 2A). We selected this task because it involves a single training session that leads to LTM and relies entirely on hippocampal function (Antunes and Biala, 2012).

After habituating the animals in the arena (Figure 2A), we exposed the four groups of animals: controls (Sc-males, Sc-females) and GluN2A-kd (2A-males and 2A-females) to the training session. We did not observe any significant difference between exploration of left and right objects in control and GluN2A-kd groups both in females and males (Figure 2B, Sc-males *p* = 0.3414, 2A-males; *p* = 0.8262; Figure 2C, Sc-females *p* = 0.1609, 2A-females *p* = 0.5823; Paired t test, Supplementary Table S1) nor in the total time exploring the objects (Figure 2D. Two-way ANOVA; Factors: Group and Sex; Group: % of variance: 0.7471, *p* = 0.6159; Sex: % of variance: 1.547, *p* = 0.4713; Interaction: % of variance 1.892, *p* = 0.4261, Supplemental Table S1).

Since animals explored both object locations similarly during the training session, we proceeded to analyze the test session. We found that both male (Figure 2E) and female (Figure 2F) control animals spent more time exploring the moved object than the object in its original location (Figure 2E, Sc-males ** *p* = 0.0037; Figure 2F, Sc-females * *p* = 0.0239; Paired t test). In contrast, neither male (Figure 2E) nor female (Figure 2F) GluN2A-kd rats showed a significant difference in the time spent exploring the moved versus the familiar object (Figure 2E 2A-males *p* = 0.6495; Figures 2F, 2A-females *p* = 0.1727; Paired t test, Supplementary Table S1). Consistently, the DI indicated that both control males and females met the learning criterion, whereas GluN2A-kd rats did not (Figure 2G, Sc-males # *p* = 0.0176, Sc-females # *p* = 0.0353, 2A-males *p* = 0.2773, 2A-females *p* = 0.2263; One sample t test. Two-way ANOVA; Factors: sex and vector; sex: % of variance: 9.461, *p* = 0.0536; vector: % of variance: 11.57, * *p* = 0.0339; Interaction: % of variance: 2.736, *p* = 0.2897, Supplementary Table S1). These results indicate that reduced GluN2A expression induces a deficit in a LTM of a spatial paradigm with one training session.

### 3.2 GluN2A-kd rats can learn a multi-session task but fail to show cognitive flexibility

Because GluN2A-kd rats did not learn a single-training-session spatial task, we asked whether they could acquire a spatial task when given multiple training sessions. To address this, we employed the BM (Figure 5A), a spatial learning paradigm that is neither emotionally nor appetitively driven and involves repeated training under low-stress conditions (Harrison et al., 2009).

**Figure 5:**
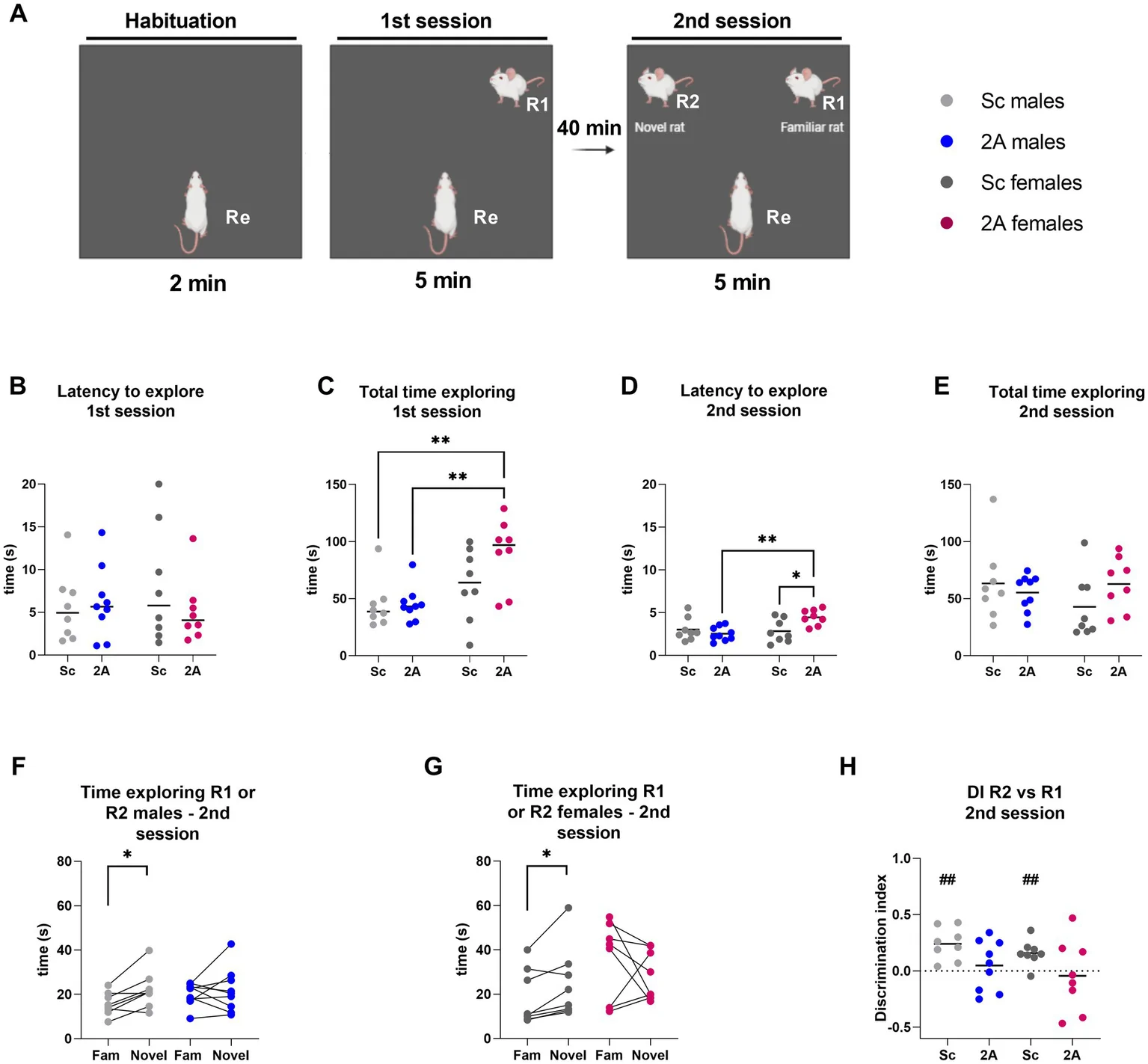
GLuN2A-kd rats were unable to discriminate a novel intruder from a familiar one in a free exploration arena. **(A)** Experimental scheme of the 2TSMT. **(B)** Latency to explore the intruder 1 (R1). The four groups (Sc-males, light gray dots; 2A-males, blue dots; Sc-females, dark gray dots and 2A-females pink dots) did not show any significant differences (Sc vs. 2A *p* = 0.4916, females vs. males *p* = 0.6872, two-way ANOVA). **(C)** Total interaction time between Re and R1. No differences were observed when compared Sc-vs 2A-rats. However, female rats explore longer the R1 (Sc vs. 2A *p* = 0.1212, male vs. female ** *p* = 0.0012, two-way ANOVA). **(D)** Latency to explore the intruder (R1 or R2) in session 2. 2A-females showed longer latencies to initiate social exploration. * *p* = 0.0333 Sc-female vs. 2A-female, ** *p* = 0.0070 2A-male vs. 2A-female; two-way ANOVA, Tukey’s multiple comparisons test. **(E)** Total exploration time for session 2. Time exploring R1 and R2 was similar across groups (Sc vs. 2A *p* = 0.5145, male vs. female *p* = 0.4770, two-way ANOVA). (F, G) Time exploring the novel (R2) vs. the familiar rat (Fam, R1) during session 2. **(F)** Sc- and 2A-males. **(G)** Sc- and 2A-female rats. Sc-males and Sc-females explored R2 significantly longer than R1 (Sc-males * *p* = 0.0118, 2A-males *p* = 0.5997; Sc-females * *p* = 0.0302, 2A-females *p* = 0.3987, paired *t* test). **(H)** DI for session 2. Sc-rats showed DI values significantly different from zero (Sc vs. 2A * *p* = 0.0132, females vs. males *p* = 0.2549; two-way ANOVA; Sc-males ## *p* = 0.0022, 2A-males *p* = 0.5265; Sc-females ## *p* = 0.0051, 2A-females *p* = 0.7068, one sample *t*-test). Sc-males, *n* = 8; 2A-males, *n* = 9; Sc-females, *n* = 8; 2A-females, *n* = 8.

Both male and female control rats showed progressively shorter latencies to locate the escape hole across training days (Figure 5B). Also, GluN2A-kd males showed reduced exploratory activity over successive training sessions (Figure 5B). In contrast, GluN2A-kd females displayed significantly higher latencies to find the escape hole from the first to the third session, showing a reduction only in the fourth training session (Figure 5B, two-way ANOVA; factors: group and time; group: % of variance: 25.39, *** *p* = 0.0004; Time: % of variance: 1.626, *p* = 0.3184; Interaction: % of variance: 0.4649, *p* = 0.4649 *** *p* = 0.0004, two-way ANOVA; * *p* = 0.0168 2A- vs. Sc-females, *** *p* = 0.0004 2A-females vs. Sc-males, *** *p* = 0.0001 2A-males vs. females; Tukey’s multiple comparison test). As expected, during the test session, control animals spent significantly more time in the target quadrant (Q1, where the escape hole had been located during training) and less time in the opposite one (Q3) (Figure 5C. Sc-males **** *p* < 0.0001; Ordinary one-way ANOVA; Q1 vs. Q3 **** *p* < 0.0001; Q1 vs. Q2 ** *p* = 0.0079; Q1 vs. Q4 ** *p* = 0.0072; Holm-Sidak’s multiple comparisons test. Figure 5D - Sc-females ** *p* = 0.0031; Kruskal-Wallis test; Q1 vs. Q3 * *p* = 0.0219; Q1 vs. Q4 ** *p* = 0.0069; Dunn’s multiple comparisons test). Similar to controls, GluN2A-kd males spent significantly more time in Q1 than in Q3 (Figure 5E, **** *p* < 0.0001; Kruskal-Wallis test; Q1 vs. Q3 **** *p* < 0.0001; Q1 vs. Q2 ** *p* = 0.0031; Q1 vs. Q4 ** *p* = 0.0020; Dunn’s multiple comparisons test). However, GluN2A-kd females spent similar time in all quadrants indicating a weak learning of the escape hole location (Figure 5F. *p* = 0.6429, Kruskal-Wallis test, Supplementary Table S1). These results suggest a sex-dependent effect of GluN2A-kd on spatial learning. Moreover, the behavioral patterns observed across training indicate that GluN2A-kd female showed impaired learning across multiple sessions and did not express the learned spatial preference in the DBM test.

Previously, it was reported that Neto1-KO mice showed GluN2A reduced levels and could acquire the MWM task but did not possess cognitive flexibility, for this reason, we examined whether our GluN2A-kd model conserve cognitive flexibility. To do this, we performed the RBM starting the day after the DBM test, using the same protocol but relocating the escape hole 180° from its original position in the previous Q3 (Figure 5A). Both control and GluN2A-kd rats showed similar latencies to locate the escape hole at the new position without significant differences between sex or injected AAV (Figure 5G, *p* = 0.1899, two-way ANOVA), which suggest that all groups of rats were able to learn the new location of the escape hole. However, GluN2A-kd females displayed markedly greater dispersion in their performance relative to controls (Figure 5G, Supplementary Table S1). These results suggest a sex-dependent exploration pattern, with a marked effect of GluN2A knockdown specifically in females.

In the RBM test session, both male (Figure 5H) and female (Figure 5I) control rats spent significant more time exploring the new target quadrant (Q3) compared to the old, opposite one (Q1) (Figure 5H *** *p* = 0.0002; Ordinary one-way ANOVA; Q3 vs. Q1: *** *p* = 0.0004; Q3 vs. Q2: ** *p* = 0.0021; Q1 vs. Q4 * *p* = 0.0435; Holm-Sidak’s multiple comparisons test) (Figure 5I **** *p* < 0.0001; Ordinary one-way ANOVA; Q3 vs. Q1: *** *p* = 0.0003; Q1 vs. Q2 ** *p* = 0.0096; Q2 vs. Q4 ** *p* = 0.0096; Q3 vs. Q4: *** *p* = 0.0003; Holm-Sidak’s multiple comparisons test, Supplementary Table S1). These results indicate that both control groups displayed LTM for the escape hole and preserved cognitive flexibility in locating the target quadrant. However, unless GluN2A-kd rats showed unexpected significant differences between Q2 and Q4 (Figures 5J,K), neither male (Figure 5J) nor female (Figure 5K) GluN2A-kd rats demonstrated a preference for the Q3 during test session (Figure 5J * *p* = 0.0347, Ordinary one-way ANOVA; Q3 vs. Q1 *p* = 0.4651; Q2 vs. Q4 * *p* = 0.0499; Holm-Sidak’s multiple comparisons test) (Figure 5K ** *p* = 0.0093, Kruskal-Wallis test; Q3 vs. Q1: *p* > 0.9999; Q2 vs. Q4 * *p* = 0.0109; Dunn’s multiple comparisons test, Supplementary Table S1) and spent similar amounts of time in all quadrants, which indicates an impairment in cognitive flexibility in GluN2A-kd rats.

Together, these data suggest that reduced GluN2A expression impairs cognitive flexibility in both sexes. Moreover, GluN2A-kd females exhibited deficits in both learning and flexibility, whereas GluN2A-kd males, despite learning the initial task, failed to update their spatially guided behavior when the escape location changed.

### 3.3 GluN2A-kd rats were able to socialize and recognize another new rat

Hippocampal NMDARs were implicated in social recognition (Zoicas and Kornhuber, 2019). Hence, we conducted a modified three-chamber exploration task (A3CST) (Yang et al., 2011) designed to assess both spatial and social recognition-association. This task involves a single training session in which animals must differentiate between an inanimate object and an unknown similar rat (R1) located under a cage, followed by a recognition phase where they choose between two conspecifics, one familiar (R1) and one novel (R2) (Figure 3A). We observed that both male (Figure 3B) and female rats (Figure 3C) whether control or GluN2A-kd, spent longer exploring the cage containing the R1 compared to the inanimate object (Figure 3B; males: Sc ** *p* = 0.0021, 2A * *p* = 0.0194, Paired t test. Figure 3C; females: Sc **** *p* < 0.0001, 2A **** *p* < 0.0001, Paired t test, Supplementary Table S1). Thus, the four groups reached the recognition criteria and did not show significant differences between them (Figure 3D. Two-way ANOVA; Factors: Sex and Vector; Sex: % of variance: 0.3278, *p* = 0.7717; Vector: % of variance: 0.04277, *p* = 0.9164; Interaction: % of variance: 0.5904, *p* = 0.6971; #### *p* < 0.0001 Sc-Males, Sc-females and 2A females; ### *p* = 0.0004 2A-males; One sample t test, Supplementary Table S1). Then, in a third session, we exposed the animals to the R1 (familiar) and to a novel, younger and same sex rat (R2, novel) (Figure 3A). The four groups spent more time exploring the novel vs. the familiar rat (Figure 3E males: Sc, * *p* = 0.0395, 2A, ** *p* = 0.0046, Paired t test. Figure 3F Females: Sc, * *p* = 0.0483, 2A, ** *p* = 0.0011; Paired t test, Supplementary Table S1) and passed the recognition criteria indicating that all the animals were able to recognize a novel or familiar congener (Figure 3G. Two-way ANOVA; Factors: Sex and Vector; Sex: % of variance: 0.002390, *p* = 0.9787; Vector: % of variance: 3.543, *p* = 0.3090; Interaction: % of variance: 0.7007, *p* = 0.6486; # *p* = 0.0152 Sc-males, ## *p* = 0.0055 2A-males, ## *p* = 0.0076 Sc-females, # *p* = 0.0153 2A-females; One sample t test, Supplementary Table S1). These results strongly suggest that the decrease in GluN2A expression after the developmental switch does not affect social recognition or social exploration.

### 3.4 GluN2A-kd rats fail to discriminate a novel conspecific in a free-exploration context

The three-chamber test is widely regarded as a reliable paradigm for assessing social interaction and recognition. However, it inherently restricts interaction, as one of the animals is confined to a fixed location within a cage. To overcome these limitations, we employed the 2TSMT (Leser and Wagner, 2015), in which the experimental subject is first habituated to a conspecific referred to a “intruder” (R1) and subsequently exposed to both the R1 and to a novel intruder (R2) in a freely accessible environment (Figure 4A).

During the first session, both male and female control and GluN2A-kd rats interacted with the R1. No differences were observed in the latency to explore between control and GluN2A-kd animals, in either sex (Figure 4B, ns two-way ANOVA; Factors: sex and vector; % of variance vector: 1.583, *p* = 0.4916; % of variance sex: 0.5398, *p* = 0.6872; Interaction: % of variance: 3.401, *p* = 0.3157, Supplementary Table S1). Male and female rats spent different amounts of time exploring the R1; furthermore GluN2A-kd females explored significantly more time than the other groups (Figure 4C, two-way ANOVA; Factors: sex and vector, % of variance vector: 5.461, ns *p* = 0.1212; % of variance sex: 27.71, ** *p* = 0.0012; Interaction: % of variance: 4.972, *p* = 0.1385; ** *p* = 0.0058 Sc-males vs. 2A-females, ** *p* = 0.0050 2A-males vs. 2A-females; Tukey’s multiple comparison test, Supplementary Table S1). Interestingly, GluN2A-kd rats exhibited a tendency toward heightened susceptibility to self-grooming (data not shown; two-way ANOVA: sex and vector; sex % of variance: 5.023, *p* = 0.2079; vector % of variance: 18.79, * *p* = 0.0192; Interaction % of variance: 0.4051; *p* = 0.7168. Non-significant differences, Tukey post-test, Supplementary Table S1). These results indicate a preserved social interaction in GluN2A-kd rats and confirm the results obtained in the first session of the A3CST. Also, they strongly suggest that females are more sensitive to GluN2A-kd than males.

During the second session, animals were allowed to explore both a familiar (R1) and a novel intruder (R2). Sc-males, GluN2A-kd males, and Sc-females exhibited similar latencies to initiate exploration. In contrast, GluN2A-kd females showed a significantly longer latency to approach a conspecific (Figure 4D two-way ANOVA; Factors: sex and vector; sex % of variance: 11.42, * *p* = 0.0339; vector % of variance: 4.832, *p* = 0.1583; Interaction % of variance: 17.01, * *p* = 0.0110; * *p* = 0.0333 Sc-female vs. 2A-female, ** *p* = 0.0070 2A-male vs. 2A-female; Tukey’s multiple comparisons test, Supplementary Table S1). On the other hand, and similarly to the first session, the total time spent exploring other animal (R1 and-or R2) was comparable across all four groups (Figure 4E, Two-way ANOVA; Factors: sex and vector; sex % of variance: 1.608, ns *p* = 0.4770; vector % of variance: 1.349, *p* = 0.5145; Interaction % of variance: 7.532, *p* = 0.1297, Supplementary Table S1). However, Sc-rats spent more time interacting with the novel rat than with the familiar one (Figures 4F,G). This difference was not observed in both females and male GluN2A-kd rats (Figures 4F,G), Sc-males * *p* = 0.0118, 2A-males ns *p* = 0.5997. Figure 4G: Sc-females * *p* = 0.0302, 2A-females *p* = 0.3987, Paired t test, Supplementary Table S1). Accordingly, DI analysis (R2 vs. R1) shows that Sc rats successfully discriminate between the novel and the familiar conspecific, while kd rats fail to do so (Figure 4H, Two-way ANOVA; Factors: sex and vector; sex % of variance: 3.630, *p* = 0.2549; vector % of variance: 18.78, * *p* = 0.0132; Interaction % of variance: 0.01818, *p* = 0.9350; ## *p* = 0.0022 Sc-males, ns *p* = 0.5265 2A-males, ## *p* = 0.0051 Sc-females, *p* = 0.7068 2A-females; One sample t test, Supplementary Table S1). These results indicate that GluN2A-kd rats fail to discriminate between familiar and novel conspecifics in a free-exploration context. This deficit suggests that GluN2A-kd induces an impaired behavioral-updating, while overall social interaction remains intact.

Altogether these results reveal that GluN2A contributes to spatially guided behavior and indicate a sex-specific effect for GluN2A-kd.

### 3.5 GluN2A-kd rats showed different exploration strategies

The results described above indicate that GluN2A-kd rats showed altered performance in spatially guided tasks. Consistent with these observations, previous work showed that kif3b-KO mice —which also exhibit reduced GluN2A levels— display abnormal exploration patterns in the Barnes maze (Alsabban et al., 2020). Based on this, we analyzed the exploration strategies of the four experimental groups in both DBM and RBM test sessions (Figure 5). We quantified the percentage of time that animals spent in each behavioral pattern (direct –D–, serial –S–, and random –R–; Figure 6A) during the test sessions we found that the four groups spent similar amounts of time in the three patterns, with no significant differences between DBM and RBM (Figure 6B; two-way ANOVA; Factors: behavioral pattern and BM phase; Sc-males: Pattern % of variance: 18.68, *p* = 0.2058; Barnes phase % of variance: 4.373e-019, *p* = 0.3089; Interaction % of variance: 1.192, *p* = 0.3032; 2A-males: Pattern % of variance: 14.13, *p* = 0.3119; Barnes phase % of variance: 9.988e-019, *p* = 0.5537; Interaction % of variance: 0.8432, *p* = 0.7714; Sc-females: Pattern % of variance: 15.53, *p* = 0.082; Barnes phase % of variance: 2.191e-08, *p* = 0.3434; Interaction % of variance: 6.133, *p* = 0.1859; 2A-females; Pattern % of variance: 12.57, *p* = 0.1190; Barnes phase % of variance: 0.003972, *p* = 0.1743; Interaction % of variance: 0.1913, *p* = 0.9745, Supplementary Table S1). Then, we decided to investigate the number of transitions that rats performed between behavioral patterns (Figures 6C,D). Sc- and 2A- males did not differ in the number of transitions in the DBM; however, in the RBM, 2A-males showed an increased number of transitions compared to Sc-males (Figure 6C Two-ANOVA; Factors: BM phase and vector. % of variance BM phase: 3.627, *p* = 0.2015; % of variance vector: 40.12, *** *p* = 0.0002; Interaction: % of variance: 1.650, *p* = 0.3849 DBM 2A-males vs. RBM Sc-males ** *p* = 0.0027; RBM Sc-males vs. RBM 2A-males **p = 0.006; Sidak’s multiple comparison test, Supplementary Table S1) In contrast, GluN2A-kd females exhibited a higher number of transitions than control females in both DBM and RBM (Figure 6D Two-ANOVA; Factors: BM phase and vector % of variance BM phase: 3.735, *p* = 0.1505; % of variance vector: 41.17, **** *p* < 0.0001; Interaction: % of variance: 0.1494, *p* = 0.7702; DBM Sc-females vs. DBM 2A-females ** *p* = 0.0047; DBM 2A-females vs. RBM Sc-females *** *p* = 0.0005; RBM Sc-females vs. RBM 2A-females * *p* = 0.0137; Tukey’s multiple comparison test, Supplementary Table S1). Consistently, the increased number of transitions in GluN2A-kd rats coincides with the task that they failed to learn (Figure 5). Therefore, we analyzed whether transition probabilities differ between groups using Markov chains (Figure 6E). We first examined global differences between DBM and RBM and found no significant effects (*p* = 0.0726 global Fisher exact test). Likewise, no differences were detected between females and males (*p* = 0.0634 global Fisher exact test). However, comparisons between Sc and 2A rats revealed significant differences (* *p* = 0.0172 global Fisher exact test). GluN2A-kd female rats were the only group that showed Random→Random transitions and did not perform Direct→Direct transitions. In contrast, Sc females were more likely to transition from Serial→Direct in DBM and Random→Serial or Random→Direct in RBM (* *p* = 0.0254, ** *p* = 0.00935, * *p* = 0.0487; Wilcoxon Test). Males, instead, differed mainly in transitions involving Serial→Direct in DBM and Random→Direct or Random→Serial in RBM (* *p* = 0.0487; * *p* = 0.0254; ** *p* = 0.00935; Wilcoxon Test). These patterns indicate that GluN2A-kd females explore the maze in a more random manner, whereas GluN2A-kd males change their strategy between DBM and RBM. In contrast, Sc animals maintain stable transition probabilities across tasks.

**Figure 6:**
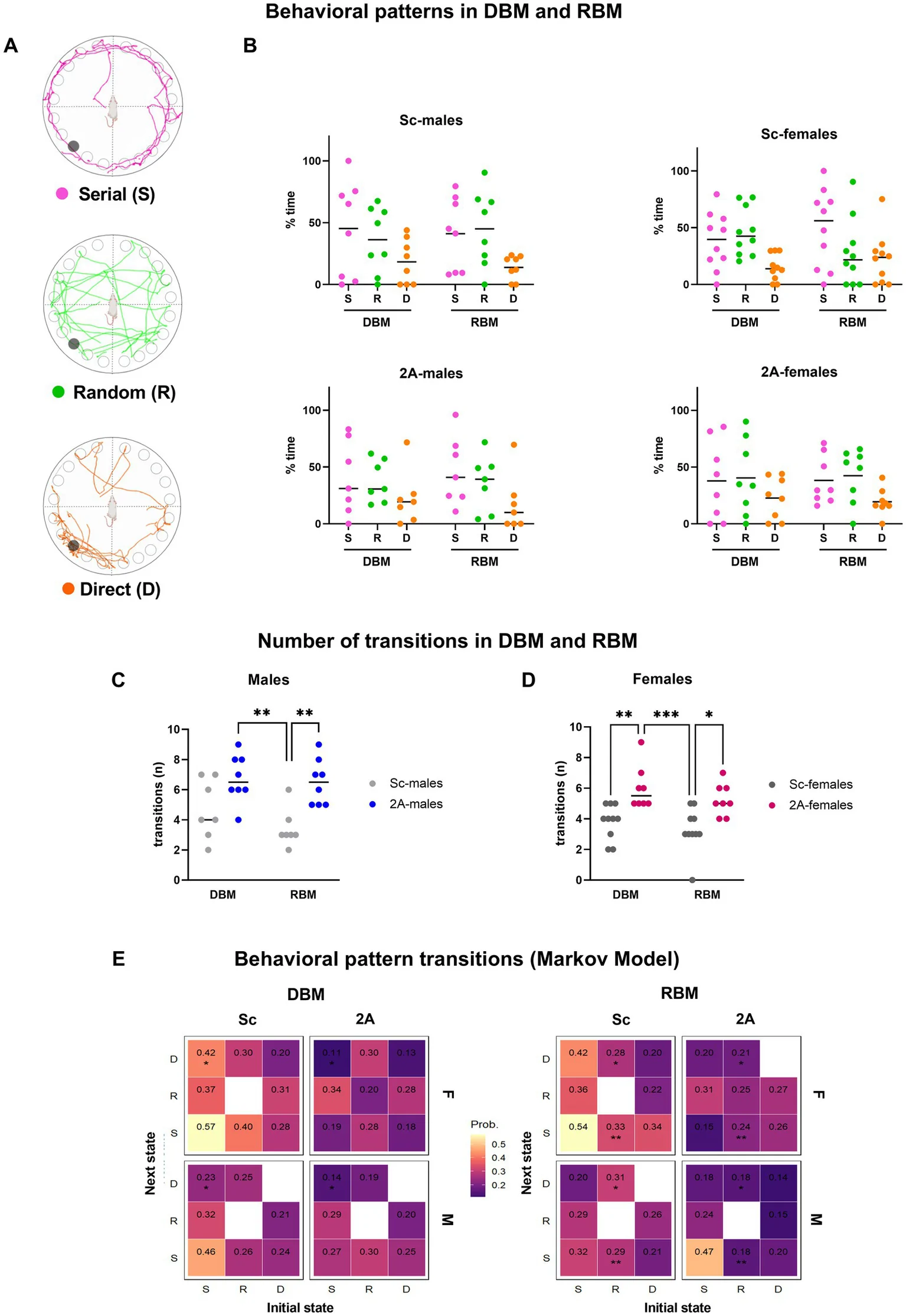
GluN2A-kd rats showed conserved exploration but altered exploration patterns. **(A)** Exploration patterns in BM tests: Direct: Heading straight to the target hole or an adjacent one (D, orange); Serial: Systematic hole-by-hole exploration (S, pink); Random: No identifiable pattern (R, green). **(B)** Percentage of time spent in each pattern for each group in DBM and RBM. All groups spent similar time in each pattern (two-way ANOVA; Factors: Behavioral pattern and BM phase; Sc-males: Pattern % of variance: 18.68, *p* = 0.2058; Barnes phase % of variance: 4.373e-019, *p* = 0.3089; Interaction % of variance: 1.192, *p* = 0.3032; 2A-males: Pattern % of variance: 14.13, *p* = 0.3119; Barnes phase % of variance: 9.988e-019, *p* = 0.5537; Interaction % of variance: 0.8432, *p* = 0.7714; Sc-females: Pattern % of variance: 15.53, *p* = 0.082; Barnes phase % of variance: 2.191E-08, *p* = 0.3434; Interaction % of variance: 6.133, *p* = 0.1859; 2A-females: Pattern % of variance: 12.57, *p* = 0.1190; Barnes phase % of variance: 0.003972, *p* = 0.1743; Interaction % of variance: 0.1913, *p* = 0.9745). **(C)** Number of transitions for male animals during DBM and RBM test sessions. There is a significant difference between 2A- and Sc-rats. Two-way ANOVA; Factors: BM phase and vector. % of variance BM phase: 3.627, *p* = 0.2015; % of variance vector: 40.12, *** *p* = 0.0002; Interaction: % of variance: 1.650, *p* = 0.3849. DBM 2A-males vs. RBM Sc-males ** *p* = 0.0027; RBM Sc-males vs. RBM 2A-males ** *p* = 0.006; Sidak’s comparison test. **(D)** Number of transitions for female rats during DBM and RBM test sessions. There is a significant difference between 2A- and Sc-rats in both sessions. Two-way ANOVA; Factors: BM phase and vector. % of variance Barnes phase: 3.735, *p* = 0.1505; % of variance vector: 41.17, **** *p* < 0.0001; Interaction: % of variance: 0.1494, *p* = 0.7702; DBM Sc-females vs. DBM 2A-females ** *p* = 0.0047; DBM 2A-females vs. RBM Sc-females *** *p* = 0.0005; RBM Sc-females vs. RBM 2A-females * *p* = 0.0137; Tukey’s multiple comparison test. **(E)** Heat map of Markov series showed that the four groups of animals display different probabilities to explore the space during BM test sessions. However, Sc-rats showed more similar strategies between DBM and RBM (DBM vs. RBM *p* = 0.0726; female vs. male *p* = 0.0634; Sc vs. 2A * *p* = 0.0172; global Fisher exact test). Comparing Sc- vs. 2A- rats in each group, we found some transitions with significantly more probabilities (Sc- vs. 2A-females Serial → Direct in DBM * *p* = 0.0254; Random → Serial in RBM ** *p* = 0.00935; Random → Direct in RBM * *p* = 0.0487; Sc- vs. 2A-males Serial → Direct in DBM * *p* = 0.0487; Random → Direct in RBM * *p* = 0.0254; Random → Serial in RBM ** *p* = 0.00935; Wilcoxon test). Sc-males, *n* = 8; 2A-males, *n* = 7; Sc-females, *n* = 10; 2A-females, *n* = 8.

Together, these findings indicate that GluN2A-kd rats display distinct exploration patterns and transition profiles, which may underlie their impaired performance in spatially guided tasks.

### 3.6 GluN2A-kd rats spatial index correlate with grin2A expression levels

To further analyze the relationship between reduced GluN2A expression and behavioral performance, we quantified GluN2A levels in male (Figure 7A, * *p* = 0.0106, Student test) and female GluN2A-kd rats (Figure 7B, * *p* = 0.033, Student test, Supplementary Table S1) and then performed correlation analyses (Figures 7C,D) between relative GluN2A levels in males and females and the DI from the A3CST (Figure 3G, purple dots at Figure 7D), and also with the DI from the 2TSMT (Figure 4H, green dots at Figure 7D), both assessing social interaction but under fixed versus freely moving conditions. We also analyzed the correlation between relative GluN2A mRNA levels and the DI from the NOL task (Figure 2G, orange dots at Figure 7D) and the DI from the novel object recognition (NOR) task, as we previously reported that GluN2A-kd rats were able to learn this paradigm (Acutain et al., 2021); (turquoise dots at Figure 7D). We included this analysis as NOL and NOR share the same training session but differ in the spatial demands of the test session.

**Figure 7:**
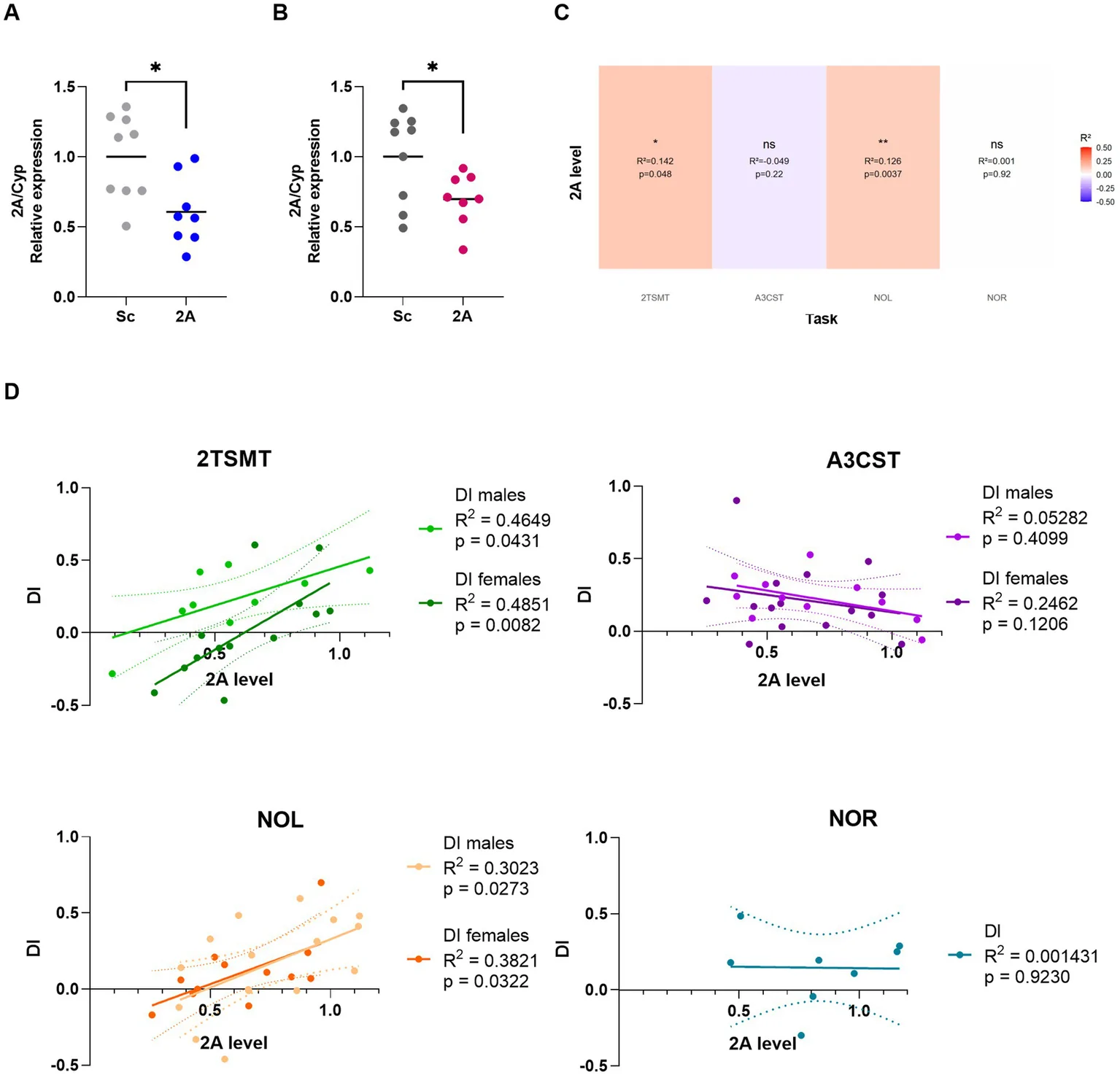
*Grin2A* expression levels correlate with DI values of tasks requiring rapid adjustments in spatially guided behavior. **(A)** Relative *grin2A* levels normalized to Sc-males for Sc-males (light gray dots) and 2A-males (blue dots). * *p* = 0.0106; Unpaired *t* test. Sc-males, *n* = 9; 2A-males, *n* = 8. **(B)** Relative *grin2A* levels normalized to Sc-females for Sc-females (dark gray dots) and 2A-females (pink dots). * *p* = 0.0333; Unpaired *t* test. Sc-females, *n* = 9; 2A-females, *n* = 8. **(C)** Heat map showing correlation levels between relative *grin2A* levels and DI values for the 2TSMT; A3CST; NOL; and NOR (Acutain et al., 2021) tasks. *R* and *p* values are indicated in the graph together with significance levels for each task. **(D)** Linear regression plots for each task: 2TSMT, green dots; dark, females *R*^2^ = 0.4851, *** *p* = 0.0082; light, males *R*^2^ = 0.4649, ** *p* = 0.0431; A3CST, purple dots dark, females *R*^2^ = 0.05282, *p* = 0.4099; light, males *R*^2^ = 0.2462, *p* = 0.1206; NOL, orange dots; dark, females *R*^2^ = 0.3821, * *p* = 0.0322; light, males *R*^2^ = 0.3023, * *p* = 0.0273; and NOR, turquoise dots, *R*^2^ = 0.001431, *p* = 0.9230. Linear regression.

We found that GluN2A expression levels correlated only with the DIs of tasks that include spatial components required for learning (Figure 7C; 2TSMT: R^2^ = 0.147, * *p* = 0.0483; A3CST: R^2^ = 0.049, *p* = 0.2196; NOL: R^2^ = 0.1289, * *p* = 0.037; NOR: R^2^ = 0.001431, *p* = 0.923; linear regression, Supplementary Table S1). These results indicate that reduced GluN2A expression is specifically associated with the performance in tasks that require spatially guided behavior.

## 4 Discussion

Understanding how molecular changes alter behavior is essential for interpreting processes relevant to neurodevelopmental and neurodegenerative diseases. In this study, we examined how reduced hippocampal expression of GluN2A affects spatial long-term memories and exploratory behavior, domains commonly altered in neuropathologies (Franchini et al., 2020; Benke et al., 2021; Zhang et al., 2025). Reducing GluN2A expression in mature hippocampal neurons selectively disrupted performance in spatially guided tasks, leaving nonspatial and social recognition behaviors largely unaffected. These results indicate that GluN2A is not uniformly required for all forms of hippocampus-dependent processes. Instead, this subunit appears to contribute to behavioral performance in tasks that require flexible spatial guidance. Furthermore, by decreasing GluN2A expression after the developmental switch, our study isolated aspects of GluN2A function that cannot be inferred from constitutive KO or developmental models and highlighted the importance of analyzing the role of GluN2A in mature circuits.

Previous work using KO mice, or indirect reductions of GluN2A levels consistently reported impairments in spatial tasks, contextual fear conditioning, or object location memory (Kiyama et al., 1998; Bannerman et al., 2008; Ng et al., 2009; Franchini et al., 2020; Gao et al., 2022; Farsi et al., 2023; Hosseini et al., 2025). However, these approaches are not able to distinguish developmental effects from mature neuronal function. On the other hand, postnatal GluN1 silencing demonstrated that NMDAR activity in DG and CA1 is essential in using spatial knowledge to select between alternative responses that arise from competing or overlapping memories and not for memory consolidation (Bannerman et al., 2012). Our findings refine this framework by demonstrating that GluN2A—rather than the complete NMDAR—is not uniformly required for performance across hippocampal-dependent tasks. GluN2A-kd rats failed in one session training spatial paradigms and showed reduced performance in tasks requiring flexible spatial guidance. These patterns suggest that GluN2A expression contributes to behavioral performance when rats must adapt their spatial behavior in changing conditions.

It is well established that spaced or repeated training can improve learning performance (Smolen et al., 2016). Moreover, some studies have proposed that hippocampal dysfunction may recruit alternative structures—particularly the infralimbic and prelimbic prefrontal cortices—through compensatory processes that support spatial learning (Zelikowsky et al., 2013). These mechanisms could help explain why GluN2A-kd males, who did not learn the NOL task, were nevertheless able to acquire the DBM across repeated sessions. In contrast, GluN2A-kd females did not show improvement across DBM training. We hypothesize that four sessions may have been insufficient for females to engage such compensatory processes, although additional molecular or circuit-level mechanisms cannot be ruled out (Zelikowsky et al., 2013; Smolen et al., 2016).

Sex differences in BM performance have been reported across strains and drug treatments (Gawel et al., 2019), and in an Alzheimer’s disease model (O’Leary et al., 2011). However, this is the first time that a sex dependent effect associated with altered NMDAR subunit expression, with females showing greater sensitivity to decreased GluN2A expression reflected in distinct exploration patterns and reduced performance at the DBM. The observed differences may arise from less efficient spatial exploration during the DBM or from behavioral patterns that limit performance under increased spatial demands. Future work will be essential to distinguish between these possibilities.

Some authors have reported sex differences in NMDAR function (Wang et al., 2010), as well as estradiol-dependent differences in behavior (El-Bakri et al., 2004). In our study, we did not observe differences in task performance between control groups; however, exploration patterns and transitions differed between control males and females, and these differences were even more pronounced in GluN2A-kd animals, confirming a greater sensitivity of females to reduced GluN2A expression and highlighting the importance of considering sex as a biological variable in studies of hippocampus-dependent behavior and NMDAR signaling.

Ng et al. (2009) previously showed that an indirect reduction of brain GluN2A levels impairs cognitive flexibility and similar deficits were reported in *kif3b*-KO mice, which exhibit decreased GluN2A expression (Alsabban et al., 2020). In our model, hippocampal GluN2A-kd also induced an impairment in behavioral flexibility. This finding was unexpected because cognitive flexibility is typically associated with cortical structures rather than hippocampal NMDAR expression (Alsabban et al., 2020; Nakazawa and Sapkota, 2020; Gao et al., 2022; Farsi et al., 2023; Herzog et al., 2023), suggesting that this function may depend on coordinated interactions between the hippocampus and cortical structures.

Impaired cognitive flexibility is currently considered a symptom of SCZ (Ragozzino, 2007; Alsabban et al., 2020; Seifried et al., 2023), raising the possibility that hippocampal GluN2A levels may contribute to the emergence of this phenotype. Importantly, the first signs of SCZ appear during adolescence or early adulthood, after the switch in GluN2 subunits has been established (Seifried et al., 2023). Consistent with our results, GluN2A variants that result in reduced GluN2A expression levels or lead to NMDAR hypofunction have been associated with increased SCZ risk (Nakazawa and Sapkota, 2020; Gao et al., 2022; Farsi et al., 2023; Herzog et al., 2023; Hosseini et al., 2025). According to the glutamatergic hypothesis of SCZ, NMDAR hypofunction may be preceded by a period of NMDAR hyperfunction and excitotoxic stress (Hu et al., 2015; Nakazawa and Sapkota, 2020). In a previous work in neuronal cultures, we showed that reduced GluN2A expression maintains neurons in an immature state with increased sensitivity to glutamatergic stimuli (Acutain and Baez, 2024). Together with the present findings, these observations support a model in which altered GluN2A expression disrupts the maturation and function of hippocampal–cortical interactions relevant to cognitive flexibility. However, additional experiments are required to confirm this hypothesis.

Hyperexcitability observed *in vitro* (Acutain and Baez, 2024) may contribute to increased seizure susceptibility observed *in vivo* (Acutain et al., 2021) in previous work. However, GluN2A-kd rats did not show any signs suggestive of seizure-related events during handling, free exploration, or social interaction in the present study. Consistent with previous findings, GluN2A-kd and control rats explored similar amounts of time, with comparable velocity or total area covered. Nonetheless, GluN2A-kd rats displayed a higher number of transitions between behavioral patterns and reduced cognitive flexibility. These features would be more consistent with altered repetitive behaviors and cognitive inflexibility observed in SCZ models (Herzog et al., 2023) than with seizure-related phenomena.

Another aspect of cognition affected in SCZ and mediated by the hippocampus is social interaction, recognition, and memory (Alsabban et al., 2020). To evaluate these domains in our model, we examined GluN2A-kd rats and found that they were able to socialize and interact with congener similarly to control animals. Previous studies have characterized sociability in GluN2A^+/−^ and GluN2A^−/−^ rodents, showing that the absence of GluN2A expression can differentially affect sociability (Alsabban et al., 2020; Bertocchi, 2021). Further experiments are needed to determine whether the absence of GluN2A during early developmental stages or the prolonged reduction of GluN2A expression is responsible for the observed deficits. In our model, both female and male GluN2A-kd rats failed to discriminate between familiar and novel conspecifics only in a free-exploration context. This deficit suggests that GluN2A expression contributes to spatially guided behavior when cues change rapidly, while leaving general social novelty and social interaction largely unaffected. However, we cannot exclude the possibility that free exploration may induce emotional arousal in the animals, thereby influencing task performance.

Consistent with the spatial hypothesis, GluN2A levels correlated with the discrimination index in paradigms that require rapid adjustments in spatially guided behavior, but not in fixed-reference spatial paradigms. This pattern supports the conclusion that reduced GluN2A expression in hippocampus selectively affects behavioral performance in tasks with more demanding spatial demands.

Altogether, our findings reveal a previously uncharacterized role for GluN2A in spatially guided behaviors and demonstrate a sex-dependent sensitivity to reduced GluN2A expression. This work advances our understanding of how NMDAR subunit composition shapes cognitive function. Moreover, it suggests that subtle reductions in GluN2A expression in mature neurons may selectively impair the spatially guided behavior with consequences for navigation and cognitive flexibility across changing conditions.

However, this manuscript has several limitations. First, the reduction of GluN2A prior to behavioral paradigms employed does not allow us to distinguish between acquisition, consolidation, and retrieval processes. Second, while cognitive deficits could be interpreted in terms of spatial representations, the present study only assesses behavioral performance and does not directly measure hippocampal neural coding. In addition, mechanistic links between reduced GluN2A expression and synaptic or physiological consequences are inferential, as knockdown was verified by qPCR and microdissection without histological validation of viral targeting or spread. Finally, relatively small group sizes per sex and condition limit statistical power.

## 5 Conclusion

In this paper, we characterize the impairment in spatially guided and cognitive flexibility resulting from decreased hippocampal GluN2A expression in both male and female rats. Together, these findings indicate that the specific knockdown of hippocampal GluN2A subunit in rats induced atypical sequencing of exploratory behaviors across spatial domains. This disruption contributes to the impaired ability of GluN2A-kd rats to solve spatially guided tasks. Notably, females appear more sensitive to the reduction in GluN2A expression, suggesting a sex-dependent effect underlying cognitive flexibility and spatially guided behavior.

## Data Availability

The raw data supporting the conclusions of this article will be made available by the authors, by request.
